# Quiescin-sulfhydryl oxidase inhibits prion formation *in vitro*

**DOI:** 10.18632/aging.101132

**Published:** 2016-12-11

**Authors:** Yi-An Zhan, Romany Abskharon, Yu Li, Jue Yuan, Liang Zeng, Johnny Dang, Manuel Camacho Martinez, Zerui Wang, Jacqueline Mikol, Sylvain Lehmann, Shizhong Bu, Jan Steyaert, Li Cui, Robert B. Petersen, Qingzhong Kong, Gong-Xiang Wang, Alexandre Wohlkonig, Wen-Quan Zou

**Affiliations:** ^1^ First Affiliated Hospital, Nanchang University, Nanchang, Jiangxi Province, The People's Republic of China; ^2^ Department of Pathology, Case Western Reserve University School of Medicine, Cleveland, Ohio 44106, USA; ^3^ Department of Neurology, Case Western Reserve University School of Medicine, Cleveland, Ohio 44106, USA; ^4^ Department of Neuroscience, Case Western Reserve University School of Medicine, Cleveland, Ohio 44106, USA; ^5^ National Prion Disease Pathology Surveillance Center, Case Western Reserve University School of Medicine, Cleveland, Ohio 44106, USA; ^6^ VIB Center for Structural Biology, VIB, 1050 Brussels, Belgium; ^7^ Structural Biology Brussels, Vrije Universiteit Brussel (VUB), 1050 Brussels, Belgium; ^8^ Hôpital Lariboisière, Service d'Anatomie et Cytologie Pathologiques, Paris, France; ^9^ IRMB -Hôpital ST ELOI, CHU de Montpellier, Montpellier, France; ^10^ National Institute of Oceanography and Fisheries (NIFO), 11516 Cairo, Egypt; ^11^ CNS, Van Andel Research Institute, Grand Rapids, MI 49503, USA; ^12^ Department of Neurology, The First Hospital of Jilin University, Changchun, Jilin Province, The People's Republic of China; ^13^ State Key Laboratory for Infectious Disease Prevention and Control, National Institute for Viral Disease Control and Prevention, Chinese Center for Disease Control and Prevention, Beijing, The People's Republic of China; ^14^ Diabetes Research Center, Ningbo University, The People's Republic of China

**Keywords:** Quiescin sulfhydryl oxidase (QSOX), prions, recombinant prion protein, protein misfolding cyclic amplification, scrapie-infected mouse neuroblastoma cells (ScN2a)

## Abstract

Prions are infectious proteins that cause a group of fatal transmissible diseases in animals and humans. The scrapie isoform (PrP^Sc^) of the cellular prion protein (PrP^C^) is the only known component of the prion. Several lines of evidence have suggested that the formation and molecular features of PrP^Sc^ are associated with an abnormal unfolding/refolding process. Quiescin-sulfhydryl oxidase (QSOX) plays a role in protein folding by introducing disulfides into unfolded reduced proteins. Here we report that QSOX inhibits human prion propagation in protein misfolding cyclic amplification reactions and murine prion propagation in scrapie-infected neuroblastoma cells. Moreover, QSOX preferentially binds PrP^Sc^ from prion-infected human or animal brains, but not PrP^C^ from uninfected brains. Surface plasmon resonance of the recombinant mouse PrP (moPrP) demonstrates that the affinity of QSOX for monomer is significantly lower than that for octamer (312 nM vs 1.7 nM). QSOX exhibits much lower affinity for N-terminally truncated moPrP (PrP89-230) than for the full-length moPrP (PrP23-231) (312 nM vs 2 nM), suggesting that the N-terminal region of PrP is critical for the interaction of PrP with QSOX. Our study indicates that QSOX may play a role in prion formation, which may open new therapeutic avenues for treating prion diseases.

## INTRODUCTION

Prions cause a group of fatal transmissible neurodegenerative disorders including Creutzfeldt-Jakob disease (CJD) in humans, scrapie in sheep and goats, bovine spongiform encephalopathy (BSE) in cattle, and chronic wasting disease (CWD) in deer and elk. To date, a misfolded prion protein (PrP^Sc^) is the only known component of the infectious pathogens. PrP^Sc^ is derived from the cellular prion protein (PrP^C^). The key molecular event in the pathogenesis of all prion diseases is the conversion of PrP^C^ into PrP^Sc^. The molecular conversion is believed to result from an abnormal unfolding/refolding process that can be triggered by mutations, seeding by exogenous PrP^Sc^ or for unknown reasons. The mechanism underlying the conversion remains unclear.

Mature human PrP^C^ is a glycoprotein encompassing residues 23-231 of the prion gene translation product containing a disulfide bond between two cysteines (Cys) at residues 179 and 214 and two consensus sites for N-linked glycosylation at residues 181 and 197 [1-5]. The protein is attached to the cell surface via a glycosylphosphatidyl inositol (GPI) anchor [6, 7]. Nuclear magnetic resonance of recombinant human PrP (23-230) revealed that the protein contains a flexible N-terminal domain from residues 23-124 and a folded C-terminal domain from residues 125-228 comprising two β-strands and three α-helices [8]. Helix 2 (α2) and helix 3 (α3), found in the C-terminal domain, are linked by the disulfide bond believed to play a role in stabilizing PrP. Therefore, it is conceivable that the structural transition of PrP^C^ to PrP^Sc^ could be mediated by the disulfide bond in PrP through changes in the redox state of the cell.

Quiescin-sulfhydryl oxidase (QSOX) has been found to catalyze the facile direct introduction of disulfide bonds into unfolded, reduced proteins with the reduction of molecular oxygen to generate hydrogen peroxide [9]. QSOX was initially found in seminal vesicle secretions and chicken egg white [10, 11]. Subsequently, QSOX has been found to be an evolutionarily conserved protein present in organisms ranging from the smallest free-living eukaryotes to humans [9, 12-15]. Moreover, QSOX has been detected in immature and mature neurons, but not in glial cells in the rat brain [16].

We investigated the effect of QSOX on the conversion of PrP^C^ into PrP^Sc^ based on its function and distribution. In the current study, we demonstrated that QSOX inhibits PrP^Sc^ formation not only *in vitro* in protein misfolding cyclic amplification, but also in scrapie-infected murine neuroblastoma cells. Furthermore, QSOX binds PrP^Sc^, but not PrP^C^, isolated from human or animal brain homogenates.

## RESULTS

### Specific interaction between QSOX and PrP^Sc^

**Figure 1:**
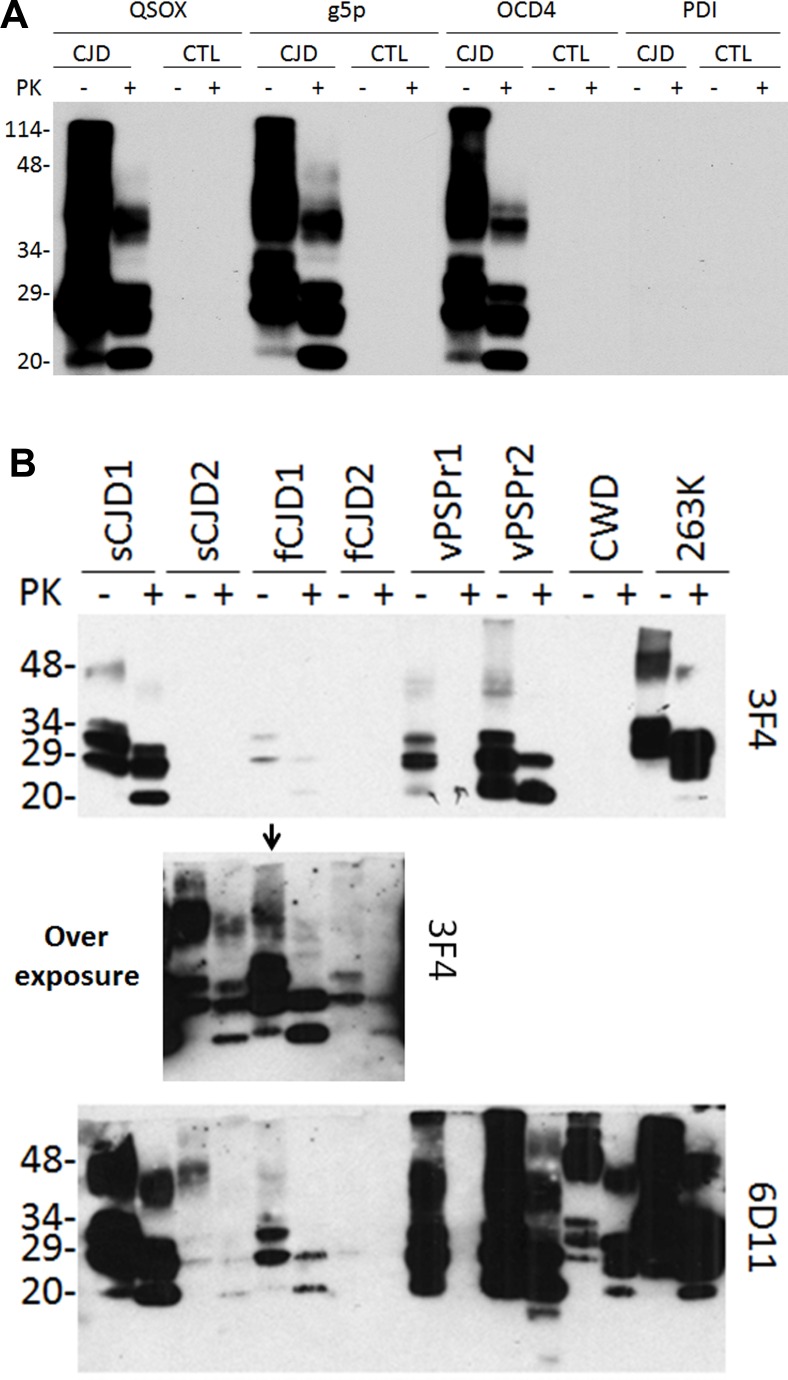
Western blotting of PrP captured by QSOX (**A**) PrP eluted from the QSOX-conjugated beads was detected by Western blotting with the anti-PrP antibody 3F4. The g5p-beads and OCD4-beads were used as controls while the PDI beads served as a negative control. Like g5p and OCD4, QSOX captures PrP only from CJD but not from non-CJD control brain homogenate. (**B**) PrP was captured by the QSOX beads from brain homogenates of two sCJD cases (sCJD1 and sCJD2), two cases with different genetic prion diseases (fCJD1 and fCJD2), two cases with VPSPr (Variably protease-sensitive prionopathy) (VPSPr1 and VPSPr2), CWD and hamster 263K. PrP^Sc^ captured from sCJD2 and fCJD2 was detectable by 3F4 only on the overexposed film (middle panel shows the overexposed part of the above blot containing samples from sCJD2, fCJD1 and fCJD2). The blots were probed with the 3F4 and 6D11 antibodies and they are a representative of three experiments.

To determine whether QSOX and PrP^Sc^ or QSOX and PrP^C^ interact, we incubated brain homogenates from sCJD or non-CJD patients with QSOX conjugated to magnetic beads. g5p- and OCD4- conjugated beads were used as positive controls while PDI (protein disulfide isomerase)-conjugated beads were used as a negative control. After elution of the captured PrP from the beads, the samples were treated with or without PK prior to Western blot analysis. Western blotting detected PrP not only in untreated but also in PK-treated samples from sCJD (Fig. 1A). In contrast, no PrP was captured by QSOX-beads from non-CJD in either untreated or PK-treated samples. As expected [17], PrP was also captured by g5p- or OCD4-beads, but not by PDI-beads. These results indicated that QSOX can specifically capture PrP^Sc^ but not PrP^C^. Notably, less than half of the PrP^Sc^ remained after PK-digestion, suggesting that more than half of the captured PrP^Sc^ is PK-sensitive PrP^Sc^.

Next, different PrP^Sc^ strains from a variety of animal and human prion diseases were examined. Two sCJD, two familial CJD, two VPSPr (Variably protease- sensitive prionopathy, an atypical human prion disease first identified by our group) [18] (one VPSPr-129MM and one VPSPr-129MV), CWD and hamster 263K were tested. PrP^Sc^ was captured from all prion-infected brains although the efficiency varied (Fig. 1B). As revealed previously [19], 3F4 mainly detected human and hamster PrP while 6D11 detected not only PrP from the two species but also CWD PrP.

### SPR analysis of interaction of various recombinant PrP molecules and QSOX

**Figure 2:**
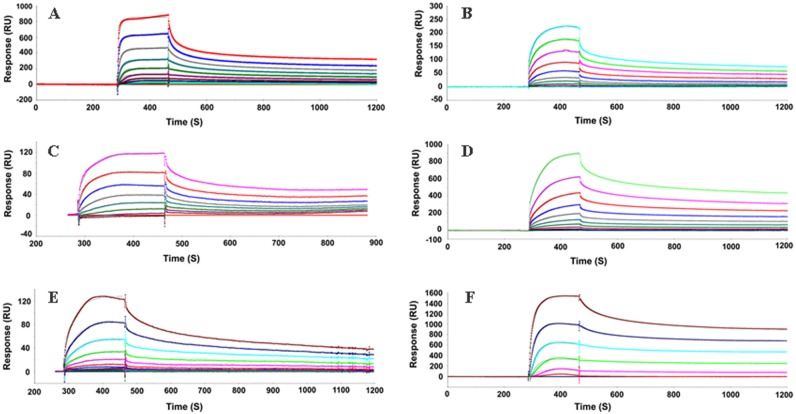
Sensorgrams of binding kinetics of immobilized QSOX to various PrP proteins Surface Plasmon Resonance (SPR) was applied as a label-free detection method to determine the binding kinetics of QSOX with various domains of PrPs. The results reveal that the equilibrium constant (KD) is relatively low for the C-terminal globular region of human or mouse PrP (472 or 312 nM) comparing to the unfolded N-terminal region of HuPrP23-145 (3.6 nM). QSOX exhibits a higher affinity for the unstructured N-terminal region than for the C-terminal folded region of PrP, suggesting that the unfolded N-terminal region of PrP is a substrate for QSOX. Interestingly, the octamer form of moPrP89-230 displays high affinity toward QSOX (1.7 nM), indicating that a misfolded form of PrP is a potential substrate for QSOX, in agreement with our *in vitro* study showing that QSOX binds to PrP^Sc^ from prion-infected human and animal brains. MoPrP23-230 was injected on immobilized QSOX (**A**), Octamer (**B**), MoPrP89-230 (**C**), HuPrP23-231 (**D**), HuPrP90-231 (**E**), HuPrP23-145 (**F**). Sensorgrams showing the evolution of response (RU, resonance units) using CM5 sensor chip performed in BIAcore 3000 instrument.

To further confirm the interaction between QSOX and different PrP forms, we used surface plasmon resonance (SPR) analysis with purified recombinant proteins. QSOX was immobilized on a CM5 sensor surface and different recombinant PrP molecules were used as analytes. Sensorgrams at several concentrations of PrP clearly showed a concentration-dependent binding to immobilized QSOX. We observed that the affinity of QSOX for both full-length MoPrP (23-230) and HuPrP (23-231) was within the nanomolar range (2 nM and 1.7 nM, respectively). In contrast, the truncated constructs MoPrP (89-230) and HuPrP (90-231), containing the folded globular domain of PrP, showed lower affinity, 312 nM and 472 nM, respectively (Fig. 2 and Table 1). When using the unfolded N-terminal region of HuPrP (23-145), the affinity was 3.6 nM, indicating that QSOX has the highest affinity for the N-terminal region of PrP. Although it had low affinity for MoPrP89-230, QSOX exhibited higher affinity for the octamer form than for the monomer form (1.7 nM and 312 nM, respectively), suggesting that there may be conformational binding sites on the octamer.

**Table 1:** SPR analysis of interaction of recombinant PrP and QSOX

Prion species	Antigen species	KD (nM)	Kon (1/Ms)	Koff (1/s)
MoPrP (23-230)	Human Qsox	2	3.34E+04	6.81E-05
Octamer (MoPrP89-230)	Human Qsox	1.7	1.09E+04	1.86E-05
MoPrP (89-230)	Human Qsox	312	3.98E+04	1.24E-02
HuPrP (23-231)	Human Qsox	1.7	1.18E+04	2.0E-5
HuPrP (90-231)	Human Qsox	472	5.68E+03	2.68E-02
HuPrP (23-145)	Human Qsox	3.6	1.32E+04	4.78E-05

### Inhibition of PrP^Sc^ amplification in cell-free protein misfolding cyclic amplification (PMCA) by QSOX

**Figure 3:**
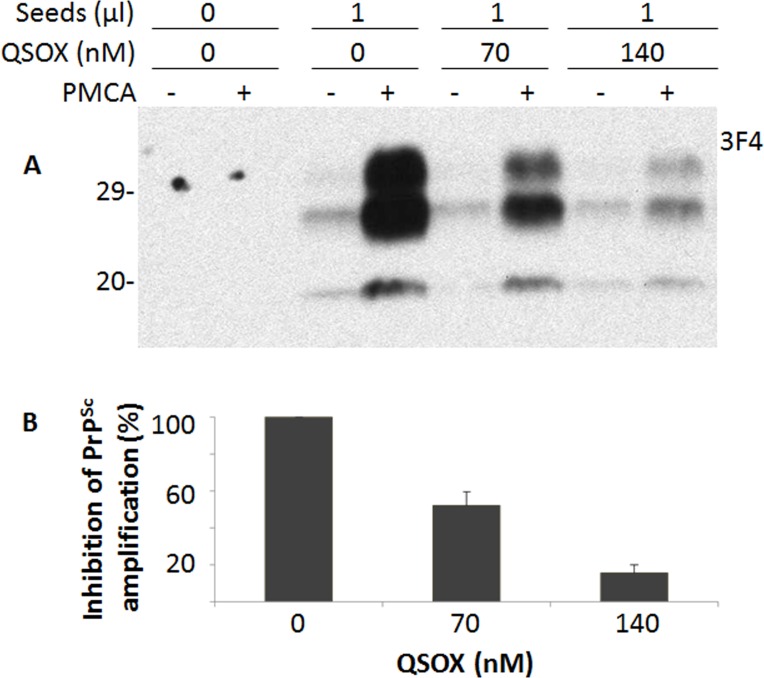
Effect of QSOX on PrP^Sc^ amplification by PMCA (**A**) Western blotting of PrP in the PMCA products after PrP^Sc^ amplification by PMCA in the presence of different amounts (0, 70 nM, and 140 nM) of QSOX for a round of PMCA. PrP^Sc^ was detected by Western blotting with the 3F4 antibody after a round of PMCA. The blot is a representative of three experiments. (**B**) Quantitative analysis of the amounts of PrP^Sc^ amplified by PMCA.

Since QSOX binds PrP^Sc^, we hypothesized that QSOX may be able to inhibit PrP^Sc^ formation *in vitro*. To test this hypothesis, we investigated the effect of QSOX on PrP^Sc^ amplification by PMCA. Different amounts of QSOX were added into PMCA reaction. Dose-dependent inhibition of PrP^Sc^ amplification was observed (Fig. 3).

### Inhibition of PrP^Sc^ amplification in prion-infected ScN2a cells by QSOX

**Figure 4:**
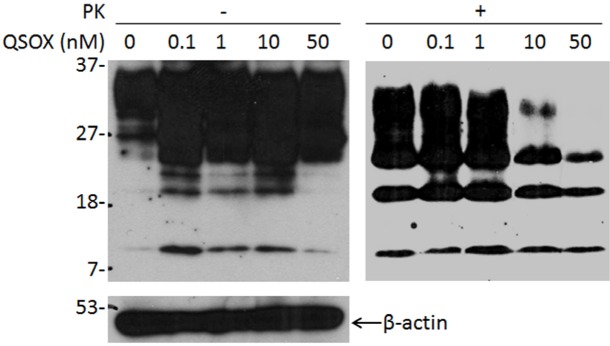
Effect of QSOX on PrP^Sc^ propagation in ScN2a cells Western blotting of PrP in ScN2a cells in the presence of different amounts of QSOX ranging from 0 to 50 nM for four days. After four days, the cells were lysed and subjected to PK-digestion at 25 μg/ml prior to SDS-PAGE and Western blotting with 6D11. The intensity of PK-resistant PrP^Sc^ is significantly decreased at 10 nM of QSOX or greater. β-actin was determined to normalize the levels of individual samples examined. The result shown here is a representative of three experiments.

We next examined the effect of QSOX on PrP^Sc^ propagation in ScN2a cells. Different amounts of QSOX were added to ScN2a cells and incubated for three days. Cell lysates were collected and treated with PK prior to detection of PK-resistant PrP by Western blotting with the anti-PrP antibody 6D11. PK-resistant PrP decreased with increasing amount of QSOX (Fig. 4), suggesting that QSOX is able to inhibit PrP^Sc^ propagation in ScN2a cells.

## DISCUSSION

Our study made the following observations. First, QSOX is able to inhibit prion propagation in both PMCA and cultured cells. Second, QSOX preferentially binds PrP^Sc^ from prion-infected human or animal brains, but not PrP^C^ from uninfected brains. Third, the affinity of QSOX for monomer is significantly lower than that for octamer. Finally, the N-terminal region of PrP is mainly involved in the interaction with QSOX. Our findings implicate the possible role of QSOX in prion formation and may provide a novel therapeutic target for prion diseases.

QSOX enzymes combine PDI-like thioredoxin (Trx) domains and ERV-like oxidase domains [12, 20-22]. The N-terminal Trx domain contains a CxxC motif that acts as the access point for reducing protein substrates [23]. There are two helix-rich domains next to the Trx domain. The domain that is closest to the C-terminus houses FAD in redox communication with a second CxxC motif [24]. The combination of Trx- and Erv-like domains may help QSOX efficiently connect the generation of protein disulfide bonds with the reduction of molecular oxygen to hydrogen peroxide [13,20]. Codding et al investigated the interaction of QSOX with model substrates in terms of introducing intra- and inter-molecular disulfide bonds into protein substrates [24]. They demonstrated that folded proteins are poor substrates of QSOX. Interestingly, QSOX was found to generate intermolecular disulfides in unstructured substrates but not in well-folded protein substrates. Moreover, it was suggested that QSOX does not appear to have a significant binding site for unfolded protein substrates while the stringent steric requirements for disulfide exchange reactions was proposed [24].

A variety of mammalian recombinant PrP molecules expressed in *E. coli* form inactive aggregates as inclusion bodies that most likely are not folded properly in the bacteria [25, 26]. Notably, co-expression of PrP and QSOX has been reported to increase solubility of recombinant human and mouse PrP [27], suggesting that QSOX may facilitate the formation of an intramolecular disulfide bond on the recombinant PrP, a process essential for proper PrP folding. However, the molecular mechanism underlying the interaction between recombinant PrP and QSOX remains unknown.

Our surface plasmon resonance study reveals that the affinity of QSOX for recombinant PrP oligomers is much higher than for recombinant PrP monomers (1.7 nM vs 312 nM), which is consistent with our *in vitro* capture assay that QSOX captures PrP^Sc^ aggregates, but not PrP^C^ monomers from human brain homogenates. However, these results are in disagreement with the above observation with other protein substrates reported by Codding et al [24], in which QSOX was found to have no significant binding site for unfolded rRNase, a fully reduced RNase. It is possible that the discrepancy may be due to different protein substrates used or different oligomeric state of substrates examined. Given the higher direct binding activity, the other possibility also needs to be examined that PrP may be one of the predominantly structured protein substrates of QSOX, with a peculiar binding site. We observed in this study that the N-terminal region of PrP is mainly involved in the interaction with QSOX, evidenced by the observation that the affinity for QSOX was much higher in full-length human or mouse PrP than in N-terminally truncated human or mouse PrP (2 nM vs 312 nM; 1.7 nM vs 472 nM). Moreover, the unfolded N-terminal region of HuPrP23-145 exhibited the same affinity for QSOX as did the full-length HuPrP23-231.

Although it is believed that the disulfide bond may be involved in stabilizing PrP^C^, the pathophysiological roles of the redox state of the cell on the disulfide bond in the structural conversion of normal PrP^C^ and pathogenicity of the pathological PrP^Sc^ remain controversial. Some experiments suggest that the conversion of PrP^C^ into PrP^Sc^ involves a temporary reduction and subsequent re-oxidation of the disulfide bond during the conformational transition [28-30]. Moreover, it is proposed that the reduced thiol may form intermolecular disulfide bonds by oxidation upon aggregation [30]. In addition, reduction is also proposed to be an important step in mediating PrP-induced membrane disruption [31,32]. On the other hand, there are several lines of evidence showing that the intact intramolecular disulfide bond in PrP^C^ is not affected during its conversion to PrP^Sc^ and that it is not necessary to have reduced thiols for the conversion, even temporarily [33-35]. Additionally, reduction and alkylation of the PrP disulfide bond has been observed to inhibit cell-free PrP conversion [36, 37], suggesting that the permanent blocking of the thiol group of PrP significantly decreases PrP^C^ to PrP^Sc^ conversion. Notably, it has been observed that the reduced forms of full-length recombinant human and truncated PrP are more readily converted into a β-sheet-rich structure by sodium chloride associated with oligomerization compared to oxidized PrP, although no monomeric β-rich structure was detected [33]. Our current study demonstrated that the PrP^C^ to PrP^Sc^ conversion is blocked by QSOX, a protein catalyst that introduces disulfide bonds into proteins, indicating that facilitation of disulfide bond formation may inhibit PrP conversion. Co-expression of recombinant PrP and QSOX is reported to prevent the aggregation of PrP, which may block the conversion of α-PrP into β-PrP.

Our previous study revealed that reduction and alkylation of the disulfide bond can render the epitopes of the 6H4 and V14 antibodies inaccessible while the epitopes of the 3F4 and anti-C antibodies remain unchanged [37, 38]. The concealment of the 6H4 and V14 epitopes located from PrP144-152 and PrP185-196 may result from a local conformational rearrangement associated with reduction and alkylation of Cys residues. Interestingly, these treatments did not abolish the binding of 6H4 to the PrP (139–158) peptide, suggesting that the blocking of the 6H4 epitope by reduced/alkylated Cys residues involves the C-terminal regions although the epitope is separated from the disulfide bond by 24 and 60 residues, respectively. The conformational rearrangement induced by reduction and alkylation could be due to intrachain van der Waals and electrostatic interactions, which have been observed between the α2 and α3 switching region and α1 in the 3D domain-swapped dimeric crystal structure [39]. In addition, we also observed that the reduction/alkylation-induced abolition of the 6H4 epitope occurs not only in PrP^C^ but also in the PrP^Sc^ molecule [38].

In conclusion, our study demonstrates a potentially new activity of QSOX (a human chaperone with thiol /disulfide oxidase activity) in the formation and conversion of prion but also suggests a new therapeutic strategy for treating human prion diseases.

## MATERIALS AND METHODS

### Materials

Proteinase K (PK) was purchased from Sigma Chemical Co. (St. Louis, MO, USA). Mouse monoclonal antibodies 3F4, which recognizes an epitope of human PrP residues 106-110 (*KTNMK*) [19, 40], and 6D11, which recognizes an epitope of human PrP residues 93-109 (*GGTHSQWNKPSKPKTNM*), were purchased from Covance (Emeryville, CA, USA). Horseradish peroxidase (HRP)-conjugated sheep anti-mouse or donkey anti-rabbit secondary antibody was purchased from Amersham Pharmacia Biotech, Inc. (Piscataway, NJ, USA). Human brain tissues were collected at autopsy and were kept frozen at the National Prion Disease Pathology Surveillance Center (Cleveland, OH, USA) at −80°C until use.

### Preparation of recombinant QSOX and protein disulfide isomerase (PDI)

The QSOX over-expression plasmid was a generous gift from Prof. Colin Thorpe. Expression and purification were performed according to the protocol described by Heckler et al [20]. The protein disulfide isomerase (PDI) plasmid was a generous gift from Dr. Joris Messens. Expression and purification were performed as previously described [41].

### Expression and purification of various recPrPs

Open reading frames encoding HuPrP(23-231), HuPrP(90-231), MoPrP(23-230), or MoPrP(89-230) were cloned in the pET-28a vector (Novagen) as an Nde1-BamH1 fragment. HuPrP(23-231), HuPrP(90-231), MoPrP(23-230), or MoPrP(89-230) were expres-sed and purified as soluble proteins according to our previous study [27]. HuPrP(23-144) was refolded from inclusion bodies according to the literature [42, 43].

### Construction of transgenes expressing human PrP-129V

The transgene constructs are based on the murine half-genomic PrP clone in plasmid pHGPRP [44]. The HuPrP-129V open reading frame (ORF) was amplified from the human genomic DNAPAC (P1-derived artificial chromosome) clone RP5–1068H6 (obtained from the Sanger Center, Cambridge, UK) with primers HRM-F (TATGTGGACTGATGTCGGCCTCTGCAA GAAGCGC) and HRM-R (CCACCTCAATTGAAA GGGCTGCAGGTGGATAC). The PCR product was digested with *Psh*AI and *Mfe*I and used to replace the corresponding 0.97 kb *Psh*AI–*Mfe*I fragment in pHGPRP to create pHGHuPrP-129V. In the resulting pHGHuPrP-129M clones, the signal-peptide sequence is still from mouse, but the rest of the PrP ORF and the first 76 bp after the stop codon are from human *PRNP* (prion protein) genomic DNA. The inserted 0.97 kb *Psh*AI–*Mfe*I fragment in pHGHuPrP-129V was then sequenced with the primers HRM-R, HRM-F, and HP306R (CATGTTGGTTTTTGGCTTACTC). One error free clone was chosen for the creation of transgenic mice.

### Generation, screening, and characterization of transgenic humanized *Tg(HuPrP-129V)Prnp0/0* mice

The 12.2 kb HuPrP-129V transgene constructs was microinjected into fertilized FVB/NJ eggs, and planted into the oviducts of pseudopregnant CD-1 mice at the transgenic mouse facility of Case Western Reserve University (Cleveland, OH) as described previously [44]. Founder pups were screened by tail DNA PCR. All founder mice that carry the transgene were bred with FVB/*Prnp0/0* mice [45] to obtain Tg mice in PrP-null background. PrP expression in the brain and other tissues of the Tg mice were determined by Western blot analysis using the monoclonal antibody 3F4 against PrP for humanized Tg mice. The Institutional Animal Use and Care Committee and the Institutional Biosafety Committee approved all of the animal experiments in this study, and the use of human brain tissues was authorized by the Institutional Review Board.

### Preparation of brain homogenates

Frozen brain tissues from patients with various prion diseases or from animals infected with prions were obtained at autopsy. Consent to use the autopsy brain tissues were obtained for each case. A 10% (w/v) human brain homogenate was prepared in 9 volumes of 1 X lysis buffer (10 mM Tris, 100 mM NaCl, 0.5% Nonidet P-40, 0.5% deoxycholate, 10 mM EDTA, pH 7.4) and was used to provide template PrP^Sc^ for PMCA. To prepare the substrate for PMCA, brains from humanized transgenic mice described above were perfused with 5% EDTA in PBS and 10% (w/v) brain homogenate from was prepared in 9 volumes of 1 X conversion buffer (0.1% SDS, 0.1% Triton X-100, 1 x PBS, pH 7.0). The supernatant was kept at −80°C for future PMCA use after centrifugation at 1,000 g at 4°C for 10 min.

### Protein misfolding cyclic amplification

PMCA was performed as described with slight modifications [44, 46]. In brief, 99 μl of brain homogenate from uninfected transgenic mouse brains was incubated with 1 μl of brain homogenate from a case of iatrogenic Creutzfeldt-Jakob disease (iCJD). After addition of designated amounts of recombinant QSOX, all samples were adjusted to equal volumes by the addition of conversion buffer (0.1% SDS, 0.1% Triton X-100, 1 x PBS, pH 7.0). An aliquot was frozen at −80°C as a control sample without PMCA and the remaining mixture was transferred into a 0.2 ml PCR tube that was placed on a microplate horn filled with water. The sample was subjected to PMCA, consisting of cycles of 30 min incubation at 37°C followed by a 40 S pulse of sonication at 60% potency for 18 h in a sonicator (Misonix Model 3000, Farmingdale, NY). To detect the amplified PrP^Sc^, 20 μl of PMCA-treated or untreated sample was digested with PK, 100 μg/ml, for 70 min at 45°C. The reaction was terminated by adding PMSF to a final concentration of 5 mM and an equal amount of SDS sample buffer, then boiling at 100°C for 10 min. A 10 μl sample was subjected to SDS-PAGE and Western blotting with 3F4.

### Scrapie-infected mouse neuroblastoma cell culture (ScN2a)

ScN2a cells seeded in six-well plates (5 × 10^5^ cells/well) containing 3 mL supplemented MEM medium were incubated with the indicated amount of recombinant QSOX for three days. Cell lysates were prepared as described previously [47]. In brief, after removal of the media, cells were rinsed three times with PBS and lysed in 1.2 ml of lysis buffer on ice for 30 min. The cell lysates were centrifuged at 1000X*g* for 10 min at 4°C to remove nuclei and cellular debris. The supernatant was incubated with 5.5 ml pre-chilled methanol at −80°C for 2 h and centrifuged at 14 000 *g* for 30 min at 4°C. The pellet was resuspended in 100 μl lysis buffer. The protein concentration of each sample was measured with Bicinchoninic Acid (BCA) reagent (Bio-Rad, Hercules, CA). Samples of equal amounts and volumes of protein were digested with 50 μg/mL proteinase K for 1 h at 37°C. Digestion was stopped with 2 μM PMSF and an equal volume of sample buffer was added and boiled for 10 min before loading onto 15% SDS-PAGE (SDS-polyacrylamide gel electrophoresis) precast Criterion gel (Bio-Rad, Hercules, CA).

### Specific capture of abnormal PrP by QSOX

Recombinant QSOX (100 μg) was conjugated to 7 × 10^8^ tosyl activated magnetic beads in 1 ml of PBS at 37°C for 20 h [48, 49]. The QSOX-conjugated beads were incubated with 0.1 % BSA in PBS to block non-specific binding. The blocked QSOX beads were stable for at least 3 months at 4°C. Capture of PrP^Sc^ by QSOX was performed as described [48, 49] by incubating S1 fractions and QSOX conjugated beads (10 μg QSOX/6 × 10^7^ beads) in 1 ml of binding buffer (3% Tween-20, 3% NP-40 in PBS, pH 7.5). After incubation with constant rotation overnight at room temperature, the PrP-containing QSOX beads were collected using an external magnetic force and all unbound molecules in the solution were removed by washing. Following three rinses in wash buffer (2% Tween-20 and 2% Nonidet P-40 in PBS, pH 7.5), the QSOX beads were resuspended in SDS sample buffer (3% SDS, 2 mM EDTA, 10% glycerol, 50 mM Tris, pH 6.8) and heated to 95°C for 5 min to release bound proteins. g5p beads, OCD4 beads, and beads conjugated with protein disulfide isomerase (PDI) were used as controls.

### Surface plasmon resonance (SPR)

Binding kinetics were determined using a Biacore 3000 (GE Health care). 2 μg QSOX was diluted in 10 mM sodium acetate, pH 5.2 and immobilized on a CM5 chip activated with EDC/NHS using a flow rate of 5 μl/min. After immobilization and blocking with ethanolamine a steady signal of 1000 RU was obtained. All kinetic analyses were run at 30 μl/min flow rate in PBS, 0.05% Tween 20, 3mM EDTA at 25°C. After each cycle, the surface was regenerated by a 60s pulse of 100 mM glycine, pH 1.5. Association rates (K_on_) and dissociation rates (K_off_) were obtained using a 1:1 Langmuir binding model (Biacore evaluation software version 4.1). The equilibrium dissociation constant (K_d_) was calculated from the ratio K_off_/K_on_.

### Sodium dodecyl sulfate-polyacrylamide gel electrophoresis (SDS-PAGE) and immunoblotting

Samples were mixed with an equal volume of 2 X SDS sample buffer and boiled for 10 min. Proteins were separated using 15% Tris-HCl pre-cast gels (Bio-Rad). Electrotransfer of the proteins from the gel to the polyvinylidene difluoride (PVDF) membranes was performed at 70 V for 2 h. The membranes were blocked with 5% non-fat milk in TBS-T buffer overnight at 4°C or 1 h at 37°C prior to incubation with antibodies. Membrane-bound proteins were probed with 6D11 at 1:6,000, 3F4 at 1:40,000, or anti-β-actin antibody at 1:6,000. The blot was then incubated with HRP-conjugated donkey anti-rabbit or sheep anti-mouse antibody at 1:6,000. The PrP bands were visualized by enhanced chemiluminescence (ECL Plus, Amersham Pharmacia Biotech, Inc., Piscataway, NJ, USA).

### Statistical analysis

Statistical significance of differences in PrP intensity was evaluated using Student's *t*-test. A difference was considered statistically significant if *p* value was < 0.05.
